# Protamine Drives Liquid and Solid Condensation of DNA and Glycosaminoglycans

**DOI:** 10.1021/acs.langmuir.5c01954

**Published:** 2025-09-22

**Authors:** Florian J. F. van der Harten, Vahid Sheikhhassani, Alireza Mashaghi

**Affiliations:** † Medical Systems Biophysics and Bioengineering, Leiden Academic Centre for Drug Research, Faculty of Science, 4496 Leiden University, 2333CC Leiden, The Netherlands; ‡ Laboratory for Interdisciplinary Medical Innovations, Centre for Interdisciplinary Genome Research, 4496 Leiden University, 2333CC Leiden, The Netherlands

## Abstract

Phase-separated biomolecular condensates play a crucial role in cellular organization. In the nucleus, phase separation regulates the assembly and function of nuclear proteins and the genome. Here, we demonstrate that protamine, an arginine-rich nuclear protein responsible for promoting double-stranded DNA (dsDNA) compaction in sperm, undergoes phase separation with single-stranded DNA (ssDNA) and induces dsDNA aggregation. The protamine-ssDNA condensates behave as viscoelastic liquid droplets and can be modulated by varying salt concentrations or by treatment with heparin, a glycosaminoglycan polyanion, which we show displaces ssDNA. Moreover, protamine-dsDNA aggregates dissolve upon exposure to heparin, leading to the formation of condensates with a distinct morphology. This observation provides a compelling example of an aggregate-to-condensate transition in polyelectrolyte systems. Notably, direct combination of protamine with heparin results in the formation of similar phase-separated liquid-like droplets, suggesting that heparin can compete with both ssDNA and dsDNA for protamine binding. We performed a comparative analysis using other positively charged proteins and negatively charged glycosaminoglycans to gain insights into the condensation behavior of protamine and heparin. Finally, we leveraged these findings to conduct a proof-of-concept analysis aimed at developing programmable biomolecular condensates for protamine-assisted nucleic acid delivery. Given that polyanionic glycosaminoglycans have been used for sperm capacitation and that DNA fragmentation is a biomarker for infertility in males, our findings offer new insights into the mechanisms of protamine-driven DNA compaction in sperm and its potential implications for reproductive medicine.

## Graphical abstract

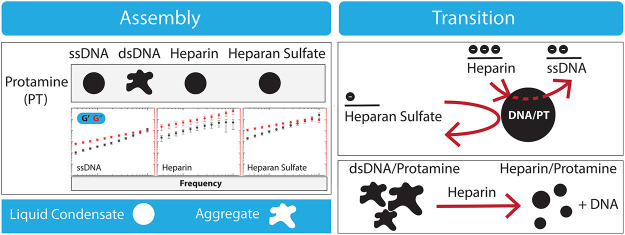

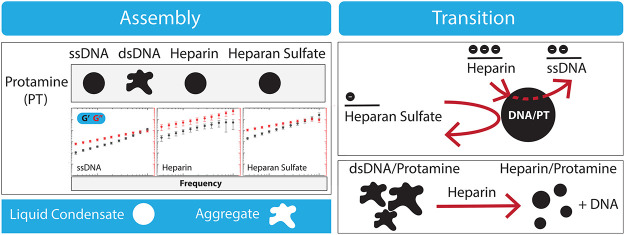

## Introduction

Nuclear organization has direct implications for cellular function.^1^ In sperm cells, the arginine-rich protein protamine binds to genomic DNA, inducing a 1 million-fold compaction that dramatically suppresses transcriptional activity.^2–6^ This tightly packed state is reversed when sperm DNA encounters the nucleoplasm of the oocyte during fertilization. Before fertilization, the sperm genome remains transcriptionally silent, with a small fraction of DNA in a single-stranded form.^7^ In contrast, oocytes and zygote cells exhibit significant transcriptional activity, which is associated with the generation of single-stranded DNA (ssDNA).^8–10^ The presence of ssDNA is also evident in infertile and immature sperms and serves as a biomarker for infertility.^7,11–13^ Therefore, understanding and regulating protamine-mediated DNA organization in both healthy and infertile sperm is of fundamental importance.

Phase separation plays a key role in biomolecular organization, allowing molecules to condense into liquid droplets that can transition into gel- or solid-like state.^14–21^ DNA molecules can condense with positively charged polypeptides, including naturally occurring histones or engineered polylysine and polyarginine.^22–26^ Protamine, which is composed of more than 50% arginine, carries a high positive charge.^27–29^ This raises the question whether it can form phase-separated droplets with DNA and thereby contribute to its organization. Furthermore, the high positive charge density of protamine may facilitate interactions with negatively charged macromolecules beyond DNA, potentially influencing its interactions with DNA.

Previous studies suggest that polyanions may affect compaction or condensation of DNA. For example, polyphosphates have been shown to affect condensation of bacterial DNA.^30^ The polyanion heparin, a negatively charged glycosaminoglycan (GAG) polymer composed of repeating disaccharide units (i.e., uronic acid and glucosamine), is shown to release DNA from complexes with positively charged dendrimers.^31,32^ Heparin is also known to interact with various DNA-binding proteins and is widely used in purifying them from DNA. In particular, heparin can bind histones, the proteins responsible for DNA organization in cells.^33^ While the most common use of heparin is as a clinical anticoagulant, it has also been shown to induce in vitro capacitation of mammalian spermatozoa.^34,35^ This raises the question of whether heparin and other similar negatively charged glycosaminoglycans, such as heparan sulfate, can affect protamine-driven DNA compaction and/or condensation.

In this article, we systematically study the phase separation and aggregation behavior of protamine with ssDNA and dsDNA, as well as the effects of treatment with glycosaminoglycans heparin and heparan sulfate. Phase-contrast microscopy, fluorescence imaging, label-free holotomography (HT), and scanning probe microscopy (SPM) are used to investigate condensate droplet formation. We then investigated whether the highly charged heparin and moderately charged heparan sulfate can displace DNA from the DNA/protamine assemblies and induce aggregate-to-condensate or condensate-to-condensate transitions. We asked whether these transitions depend on the sequence of the DNA and are driven by electrostatic interactions. Finally, we discuss the implications of these findings in the context of reproductive medicine and bio-inspired engineering of protamine-assisted delivery systems.

## Materials and Methods

### Materials

All experiments were performed on TPP Cell Culture dishes 40 mL (9.2 cm^2^), and all chemicals were purchased from Sigma-Aldrich unless otherwise specified. All DNA constructs were custom-ordered from Integrated DNA Technologies (IDT; Coralville, IA, USA). UltraPure Salmon sperm DNA was purchased from Thermo Fisher Scientific (Waltham, MA, USA). A 40-nucleotide custom hairpin DNA (5′-TTTTT­TGCGC­GCGCT­TTTTT­TTTTT­TGCGC­GCGCT­TTTTT-3′), designed to form a short stem-loop structure, was also ordered from IDT. The fluorescently labeled ssDNA (fdT40) was labeled with TYE-665 at its 5′ end. The fluorescently labeled protamine (fPT) was labeled with FITC (FITC-protamine; Xi’an Ruixi Biological Technology Co., Ltd., Xi’an, China). The 40-nucleotide random-sequence dsDNA (RS40) was purchased as a double-stranded construct with one strand having the following sequence: 5′-GAGAA­TGCGT­GACC­TTTGA­GAATA­AAGG­TCAC­GCGA­TGAA-3′.

The working buffer consisted of 50 mM HEPES (H4034) with 150 mM KCl (P5405) and 1 g/L NaN_3_ (S2002) unless specified otherwise. 50 g/L crowding agent Ficoll PM 70 (F2878) was added to mimic the cellular environment.

### In Vitro Droplet Assays

The methods for dish passivation and sample preparation were adapted from earlier research.^20^ The dishes were passivated for 30 min using 800 μL of 1% (w/v) bovine serum albumin (A2153). After 30 min, the dishes were washed 3 times using 800 μL of deionized water. The dishes were then air-dried in a dehydrator at 30 °C for 4 h, after which they were ready for use. First, a bulk droplet containing 60 μL of the working buffer was placed in a passivated dish. Next, the negatively charged component, which consisted of either heparin sodium salt from porcine intestinal mucosa (H3393), heparan sulfate sodium salt from bovine kidney (H7640), or one of the DNA constructs, was injected into the bulk droplet. Then, the positively charged component, which was either protamine from salmon (P4005) or polylysine (P2658), was also injected into the bulk droplet. Finally, the entire bulk droplet containing all components was mixed by gently pipetting up and down 10 times using a micropipette set to 40 μL and left to settle for 4 h before further analysis.

### Glycosaminoglycan Treatment

Samples are prepared as described in “in vitro droplet assays”. After 2 h, images were captured. Immediately thereafter, the GAG treatment molecule (either heparin sodium salt from porcine intestinal mucosa (H3393) or heparan sulfate sodium salt from bovine kidney (H7640)) is gently injected into the liquid phase of the bulk droplet and left for 2 h. Images were captured after 2 h.

### Phase Contrast Microscopy and Image Analysis

Phase contrast microscopy images were recorded using a Nikon Ti-2 Eclipse microscope equipped with a 20×/0.4 objective. The Z-stack was adjusted manually to ensure focus on the surface of the dish, and the bulk droplet is manually placed in the middle of the frame. For image analysis, Fiji ImageJ (version Java 1.8.0_345) was used. For the condensate maps, square sections of 300 × 300 pixels were cropped from the middle of the sample and provided with a scale bar. For particle size and circularity analysis, the entire sample was uploaded to ImageJ as a TIFF file. For particle thresholding, the color channels were split. The green channel was thresholded (200–255) to isolate the condensate particles. After thresholding, particles were analyzed for area and shape descriptors using in-built ImageJ functions (including holes, size 0-infinity, circularity 0-infinity). The results were exported to the Origin 2025 system for further processing.

### Fluorescence Microscopy and Image Analysis

For fluorescence experiments, 1% TYE-665-labeled 40-nucleotide poly-T ssDNA (5TYE665, purchased from Integrated DNA Technologies) or 5% Fitc-Protamine (from Ruixibiotech) are used. The images are taken with a Nikon Ti-2 confocal microscope using a 20×/0.75 objective with the following settings: laser intensity = 1.5, gain = 30 for fdT40, gain = 60 for fPT, size = 512 × 512, and zoom = 6.89. The fluorescence intensity is measured by calculating the *corrected total droplet fluorescence* (CTDF).^36^ Biomolecular condensate droplets were selected manually in ImageJ and measured for the mean gray value (MGV), integrated density, raw density, and area. For each sample, five fluorescent droplets and five corresponding background positions (without a fluorescent signal) close to the droplets were selected. CTDF was calculated by using the following equation:

CTDF=integrated density−(area×mean background fluorescence) 1

Fold change in CTDF compared to the untreated condition was used to quantify the displacement effect from the treatment compounds. All statistical analyses were performed in Origin 2025. Treatment effects were compared from CTDF values using the Wilcoxon Signed-Rank test (α = 0.05, *p*-value two-tailed, exact value).

### Holo-tomographic Microscopy

Holo-tomographic microscopy (HTM) was performed on the 3D Cell-Explorer Fluo (Nanolive, Ecublens, Switzerland) using a 60× air objective (NA = 0.8) at a wavelength of λ = 520 nm (Class 1 low power laser; sample exposure, 0.2 mW/mm^2^) and CMOS Sony sensor, with quantum efficiency (typical) 70% (at 545 nm), dark noise (typical) 6.6 e-, dynamic range (typical) 73.7 dB, field of view 90 × 90 × 30 μm, axial resolution 400 nm, and maximum temporal resolution 0.5 3D RI volume per second. Acquired refractive index images were processed with built-in software (Steve).

### Rheological Analysis Using Scanning Probe Microscopy

We used the recently developed SPM-based rheology approach developed by Naghilou and co-workers.^20^ A JPK CellHesion 200 (Bruker, Germany) was used in combination with a Nikon Ti-2 Eclipse microscope (20×/0.4 objective) to acquire real-time images from the sample. The cantilever was passivated with 1% Pluronic (P2443) for 30 min to prevent adhesion of the droplets on the tip. Samples were prepared as described in “In vitro droplet assays”. A μL portion was carefully taken from the dilute phase in the bulk droplet and placed on the cantilever in the JPK CellHesion 200 to avoid disturbance by air bubbles when entering the bulk droplet. The JPK CellHesion 200 was then placed onto the sample and left for 15 min to achieve thermal equilibration. This period was lengthened by 10 min if horizontal or lateral deflection of the laser was not stable yet. After thermal equilibration, the measurements were made by indenting the condensate droplets with the tip of the cantilever and harmonically driving the head for 15 cycles at different frequencies with forces of 0.4 nN, a velocity of 1 μm s^–1^, and amplitude of 40 nm to obtain head height oscillation *h*(*t*) and the resulting deflection oscillation *F*(*t*). Prior to the measurements, calibration was performed as described by Naghilou and co-workers^20^ and was used to conduct rheology data analysis using the code available through this study.

### Delivery Vehicle Experiment

First, a bulk droplet containing 60 μL of working buffer was placed on a passivated dish. Then, 30 μM heparan sulfate and 60 μM protamine were injected into the bulk droplet and gently mixed by pipetting up and down 10 times using a micropipette set to 40 μL. After mixing, the components were left to settle for 2 h. Thereafter, 30 μM of fluorescently tagged dT40 was gently injected to the dilute phase of the bulk droplet and left to settle for 2 h, after which the sample was imaged. Immediately after imaging, 30 μM heparin was gently added to the dilute phase of the bulk droplet as release compound and left to settle for 2 h. Finally, image capture and quantification were performed using the method described in “fluorescence microscopy and image analysis”.

## Results

### Protamine Forming Phase-Separated Condensates with ssDNA

To determine whether protamine and ssDNA undergo phase separation under physiological salt (KCl, 150 mM) and pH (7.6) conditions, an in vitro droplet assay using salmon-derived protamine and 40-nucleotide poly-T ssDNA (dT40) as a model system was conducted. The resulting condensate formation map, shown in Figure 1A, demonstrates that protamine and dT40 do phase separate into condensate microdroplets. Droplet size is dependent on concentration as higher concentrations yielded larger droplets across the phase space. Combining 60 μM protamine with 30 μM dT40 yielded the largest number of droplets, and this condition was chosen for further analysis. As depicted in Figure 1B, the droplet size distribution was fitted to an exponential curve:

$$y=a+be^{cx}$$ 2

The exponential curve closely fit the data, thereby revealing a formation mechanism through fast nucleation, followed by droplet coalescence.^37^ The droplets had an average circularity of 0.94 ± 0.1 (*N* = 8750), indicating a strong tendency to form round droplets within the system (inset of Figure 1B). The protamine-dT40 model system was also tested under varying KCl and MgCl_2_ concentrations, demonstrating its dependency on charged interactions (Supporting Information Figure S1). Another ssDNA construct with different base composition (40-nucleotide poly-A) was also characterized and clearly formed similar phase separated droplets (Figure S2).

**Figure 1:**
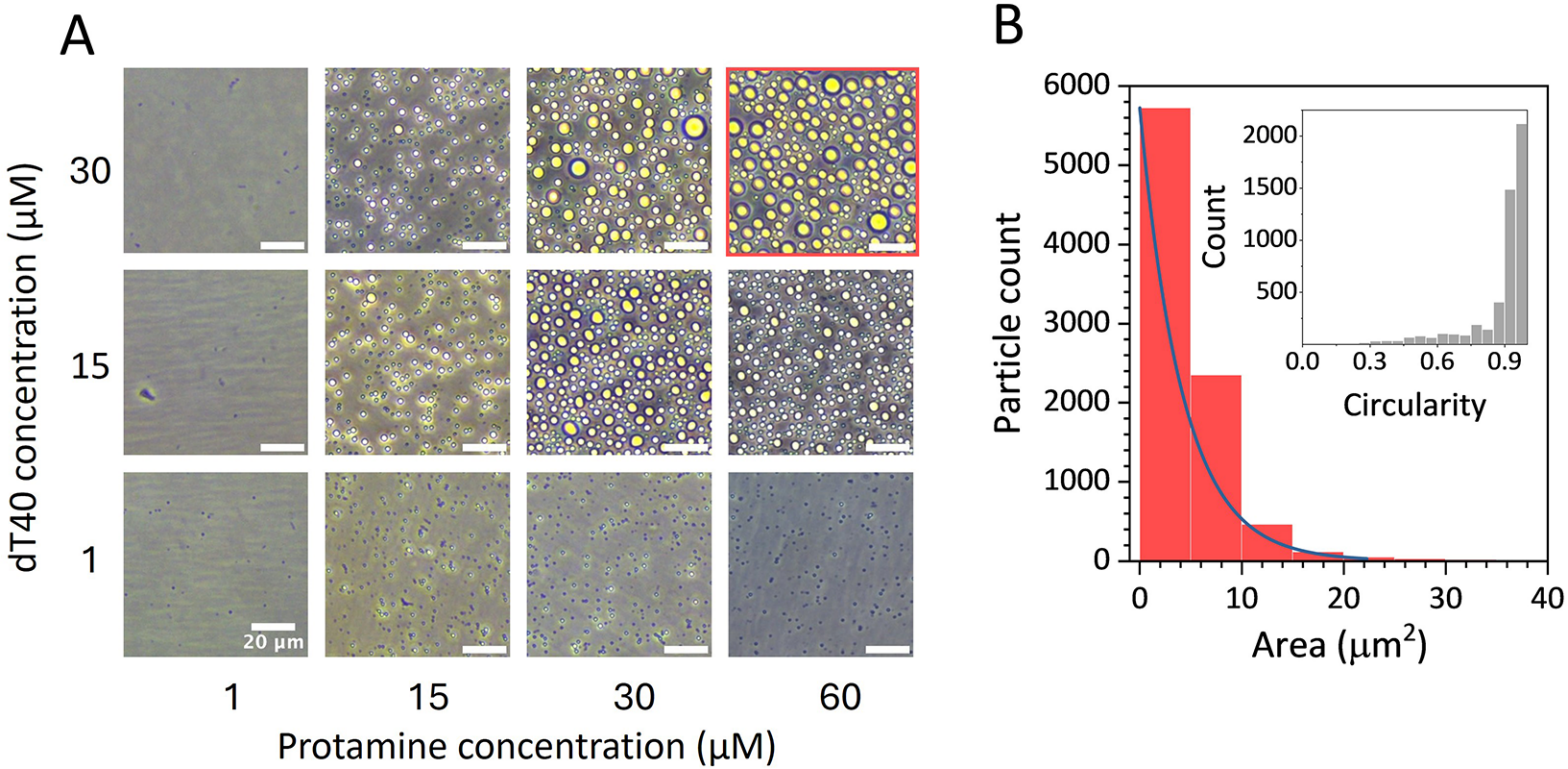
Separation of protamine and dT40 phase to form round condensate droplets. (A) Phase contrast microscopy images from in vitro droplet assay of protamine-dT40 condensates (pH = 7.6). (B) Size distribution analysis of the droplets fitted by an exponential (*y* = *a* + *b*e^*cx*^) curve. Inset: distribution of circularity values. The analysis was performed on droplets formed by 60 μM protamine with 30 μM dT40 (red outline in the condensate formation map). Scale bars are 20 μm.

### Heparin Displacing ssDNA from Protamine-DNA Condensates, Altering Droplet Morphology

We next treated the condensate droplets with GAGs to investigate the possible competition between GAGs and protamine for DNA binding. The model system consisted of protamine-dT40, which was treated with 30 μM heparin or 30 μM heparan sulfate, with an injection of working buffer serving as the control. Phase contrast microscopy images from the in vitro droplet assays (Figure 2A) show that the protamine-dT40 system is stable when untreated and upon addition of the buffer. However, the addition of heparin greatly alters the droplet morphology and causes the formation of “amorphous” condensates with reduced circularity. The system appeared less stable due to the observation of small, moving droplets. Treatment with heparan sulfate does not seem to cause any effect from the phase contrast microscopy images. Fluorescently tagged dT40 (fdT40) was used to visualize the effects of GAG treatment on protamine-dT40 binding (Figure 2B). CTDF and fold change across treatments are plotted in Figure 2C. Treatment with heparin caused a significant decrease in CTDF (*p* < 0.0001). An injection of working buffer serving as control had no significant effect (*p* = 048871). Similar treatment with heparan sulfate slightly reduced CTDF too (*p* = 0.03015), although the effect was minor compared to that with the heparin treatment. To visualize the heparin treatment effect across a time scale, phase contrast microscopy images were taken from the same area within the condensate sample every 15 min after addition of the treatment, for 2 h, shown in Figure 2D,E. Heparin treatment clearly alters droplet morphology over time, causing the formation of irregular condensate droplets and particles with active motion within the sample. This suggests that heparin is effectively displacing ssDNA from the condensates, which is further supported by the fluorescence results in Figure 2B,C. Heparan sulfate does not affect droplet morphology and does not affect the stability of the protamine-dT40 condensates, underscoring the crucial role of charge density in governing molecular interactions (Figure 2D).

**Figure 2:**
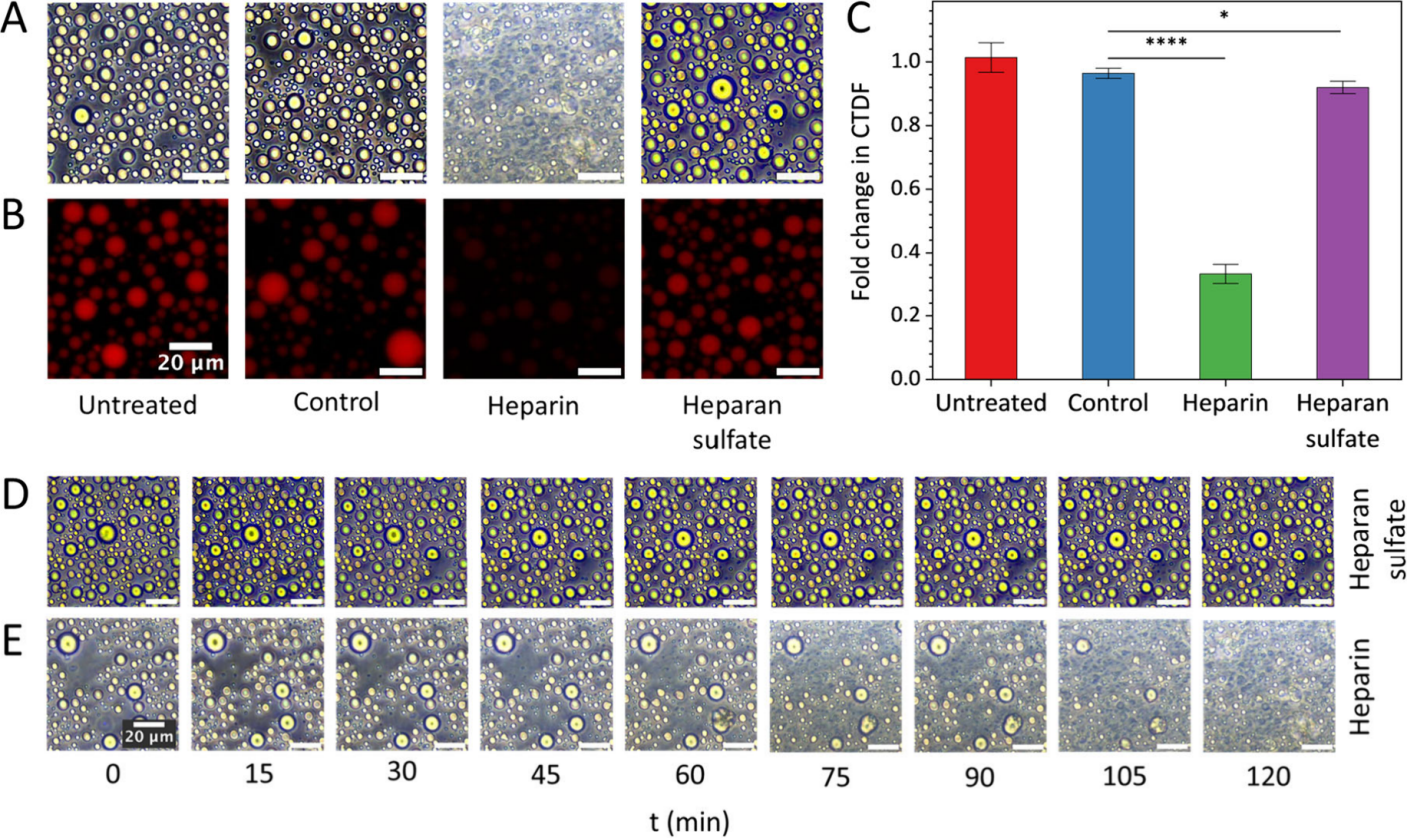
Heparin treatment displacing ssDNA from condensates with protamine, whereas heparan sulfate showing limited effect. (A) Phase contrast microscopy images from in vitro droplet assay containing protamine-dT40, treated with GAGs heparin and heparan sulfate, revealing the effect of heparin to disrupt morphology in the system. (B) Fluorescence imaging of GAG treatment effect on protamine-fdT40 condensates revealing displacement of fdT40 from condensates. (C) Fold change in CTDF showing that heparin significantly (*p* < 0.0001) displaces fdT40 from condensate droplets, whereas heparan sulfate (*p* = 0.03015) and the control (0.48871) do not. *p*-value style: two-tailed, exact-value, GP. *p*-values derived from Wilcoxon Signed-Rank Test, *N* = 15. (D) Phase contrast microscopy images from in vitro droplet assays containing protamine-dT40. 30 μM heparan sulfate is added at *t* = 0, which does not affect morphology over time. (E) Phase contrast microscopy images from in vitro droplet assays containing protamine-dT40. 30 μM heparin is added at *t* = 0, which gradually deteriorates the existing morphology and induces the formation of amorphous droplets within the system over time. Scale bars represent 20 μm. The reduced contrast observed after heparin treatment is due to changes in the chemical composition of the droplets and their environment, which alter their optical properties.

### Protamine Forming Solid-Like Aggregates with dsDNA, Which Can Be Dissolved with Heparin Treatment

To determine whether protamine and double-stranded DNA (dsDNA) can form phase-separated droplets, another in vitro assay was conducted. Protamine and 40-nucleotide poly-AT dsDNA (AT40) were mixed at different concentration ratios under physiological salt concentration (KCl, 150 mM) and pH (7.6) to construct a phase diagram (Figure 3A). Intriguingly, we observed that dsDNA does not form droplets but forms aggregated clusters with AT40. Protamine and AT40 form small, aggregated clusters at lower concentration (<15 μM) that devolve into 3D, aggregated network structures at higher concentrations (>15 μM). This behavior is in sharp contrast with the aforementioned protamine/ssDNA assembly and is similar to polylysine assembly with single-stranded and double-stranded DNA (Figure S3). Interestingly, a 40-nucleotide poly-T with short inserts capable of inducing secondary structure formation (hairpin), featuring both ssDNA and dsDNA regions, shows a coexistence of condensates and aggregates (Figure S4), consistent with the observed ability of DNA to adopt a range of states depending on its structure and folding.

**Figure 3:**
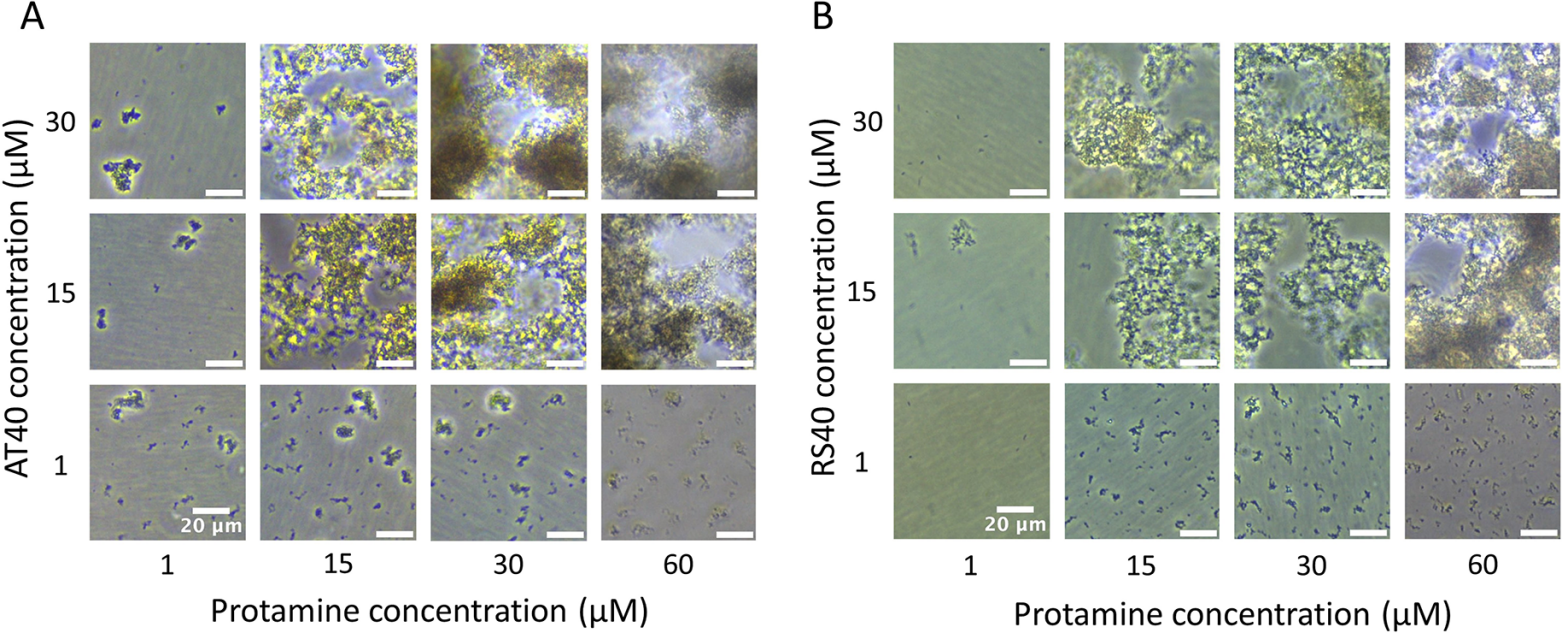
Protamine and dsDNA forming solid-like aggregates. (A) Phase contrast microscopy images of 12 different concentration ratios of protamine and AT40. (B) phase contrast microscopy images of 12 different concentration ratios of protamine and RS40. Scale bars represent 20 μm.

To test whether this observation is sequence-specific or not, we performed a similar analysis using a double-stranded DNA of a random sequence of length 40 nt (RS40),^38^ with 45% GC content. Similar to AT40, solid-like aggregates were observed in protamine-RS40 samples (Figure 3B). Motivated by earlier findings on the effect of magnesium on DNA,^30,39–41^ in vitro droplet assays were also conducted with different MgCl_2_ concentrations, where no qualitative effect (e.g., condensate formation) was observed in protamine/dsDNA phase behavior across the concentrations tested (Figure S5). To further probe the sequence-dependency of protamine/dsDNA aggregation behavior, we exposed Salmon sperm DNA to protamine at varying concentration. This analysis revealed dramatic condensation of sperm DNA through an aggregation-like process, with no apparent formation of condensate droplets (Figure S6).

To assess whether GAGs were able to displace dsDNA from protamine, an experiment using an injection of 30 μM of both GAGs was conducted. An injection of working buffer served as a control. As shown in Figure 4A, treatment with heparin completely dissolved the solid-like aggregates of protamine-AT40 and caused the formation of condensate microdroplets (diameter ∼ 1 μm). The dissolution of the aggregates further indicates that heparin is capable of displacing protamine from DNA. Similar aggregate dissolution was observed in protamine-RS40 samples, but significantly more microdroplet-like structures formed. The different observations suggest sequence-dependent protamine/DNA binding dynamics, which has been confirmed in earlier studies.^13,27^ In Figure 4B–D, the effect of the heparin concentration on the formation of microdroplets was further investigated. The formation and quantity of microdroplets was dependent on heparin concentration and DNA sequence. Heparan sulfate was not effective at dissolving aggregation but slightly dispersed the aggregates (Figure 4A), suggesting that heparin induced disaggregation is partly driven by electrostatic interactions. In Figure 4C,D, fluorescently labeled protamine (fPT) is used to visualize treatment effect. Treatment with heparin causes substantial dispersion of the fluorescent molecules and dissolution of aggregation. We further extended this analysis using sperm DNA, which features a wide range of sequences. The results confirmed the general finding that protamine induces DNA condensation through an aggregation-like concentration-dependent mechanism, which can be reversed by heparin treatment (Figure 4E).

**Figure 4:**
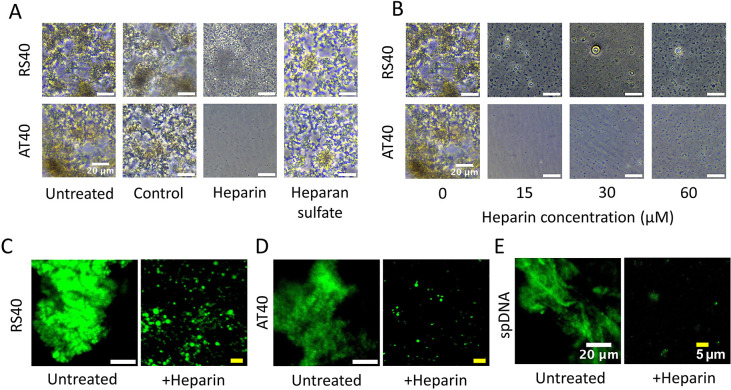
Protamine-dsDNA aggregation dissolving after heparin treatment. (A) Phase contrast microscopy images from in vitro assay containing protamine and AT40 or RS40 upon treatment with GAGs. An injection of working buffer served as control. (B) Phase contrast microscopy images from in vitro assay containing protamine and AT40 or RS40 upon treatment with varying concentrations of heparin. Scale bars represent 20 μm. (C–E) Fluorescence images of samples formed by mixing fluorescently labeled protamine with DNA constructs, followed by heparin treatment. RS40 and AT40 samples clearly showed disaggregation and a transition from aggregates to the formation of round droplets. Similarly, the sperm DNA (spDNA) formed aggregates upon exposure to protamine, which subsequently dissolved following heparin treatment. In all three cases, image gain was adjusted to improve visualization of the formed droplets.

### Protamine Forming Condensate Microdroplets with Heparin or Heparan Sulfate

To further elucidate the binding of protamine *and* heparin/heparan sulfate, phase diagrams were constructed for protamine with heparin and protamine with heparan sulfate. The results are shown in Figure 5A,B. Both heparin and heparan sulfate undergo phase separation with protamine and form condensate droplets. Phase contrast microscopy revealed the formation of larger condensate droplets in concentrations > 15 μM and smaller microdroplets in concentrations < 15 μM. The entire phase diagram for heparan sulfate is shown in Figure S7.

**Figure 5:**
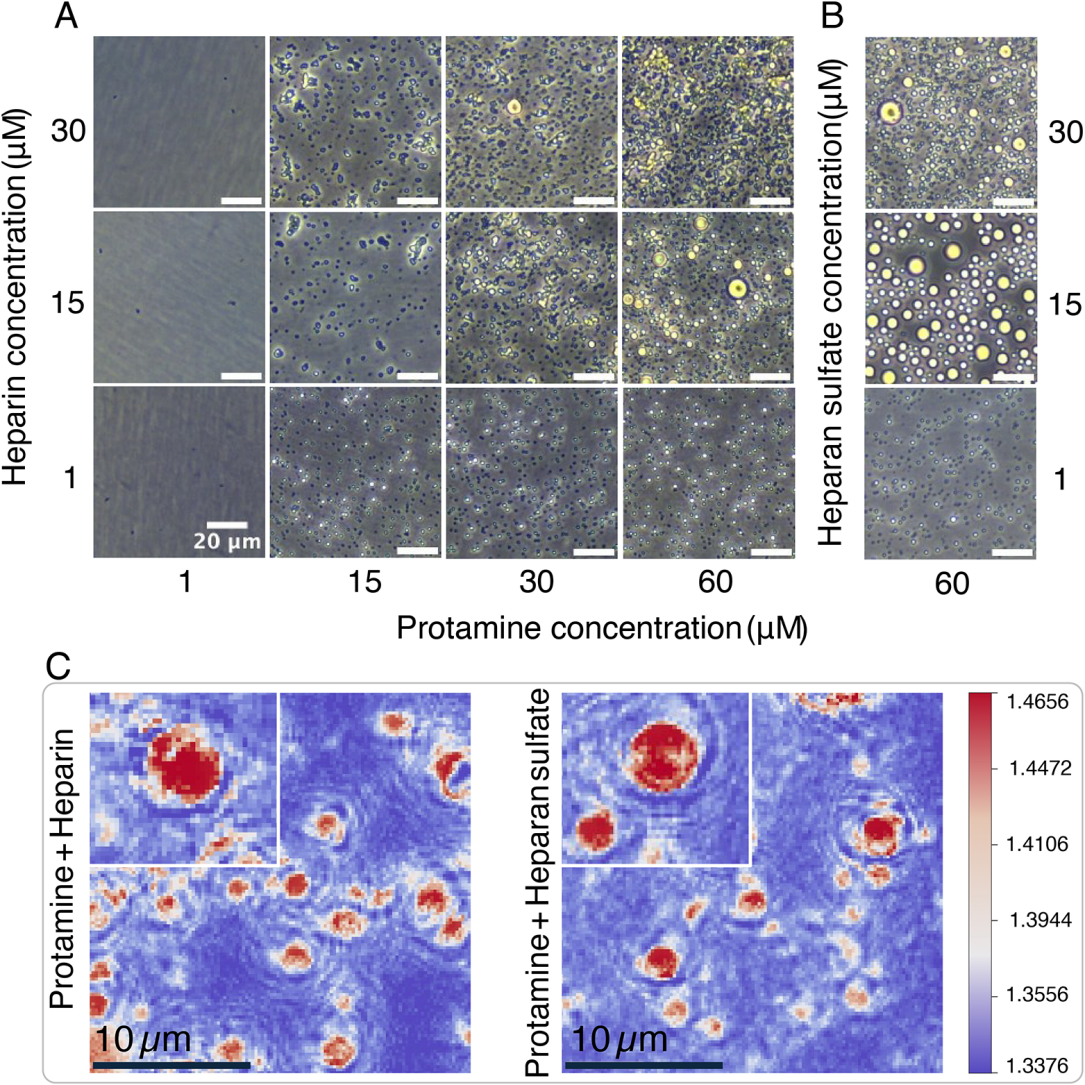
Protamine phase separation with heparin (A) and heparan sulfate (B) and formation of condensate droplets. Scale bars represent 20 μm. (C) Holotomography imaging revealing that smaller objects observed within the sample are droplets. By comparing refractive index (RI) intensities, we confirmed that these smaller features share the same RI characteristics as larger, clearly identifiable droplets (insets and top left corners). Scale bars represent 10 μm.

Given that the large and small condensate droplets appeared differently under phase contrast microscopy, likely due to optical effects, we decided to characterize these objects using a complementary, label-free approach. Label-free refractive index (RI) imaging through holotomography microscopy (HTM) was then used to characterize the objects that were observed in the samples with protamine and heparin or heparan sulfate (Figure 5C). Our data revealed that the observed small particulate objects have a peak refractive index of >1.46, similar to the larger condensate droplets. Therefore, it can be concluded that the smaller particles are also condensates with a similar chemical composition. We note that these microdroplets are rare in ssDNA/protamine samples but form droplet assemblies in heparin and heparan sulfate condensates. This observation suggests that ssDNA condensates readily fuse, whereas fusion dynamics are relatively slow in glycosaminoglycan condensates. The reduced propensity for fusion can be attributed to the lower tendency of the polymer network to rearrange and to the surface properties of the condensates.

### Protamine-Based Condensates Behaving As Viscoelastic Liquids, with Distinct Moduli for ssDNA and GAGs

We next performed SPM-based rheology to confirm that the observed condensate-like structures are indeed liquid droplets. To determine the viscoelastic properties of protamine-based condensate systems, samples were prepared using 60 μM protamine with either 30 μM dT40, 30 μM heparin, or 15 μM heparan sulfate. The samples were indented with a hemispheric cantilever and harmonically oscillated at 15 different frequency cycles, as described in Materials and Methods (Figure 6A–C). The storage (elastic) modulus *G*′ and loss (viscous) modulus *G*″ were obtained and are shown in Figure 6D–F. Protamine-dT40 condensates behave as viscoelastic liquids with viscoelastic moduli lower than those of protamine-GAG condensates. The viscoelastic moduli of protamine-dT40 condensates are nearly 2 orders of magnitude lower than protamine-GAG condensates, indicating distinct modes of protamine interactions for DNA and GAGs. The distinct mechanical profiles align with the observed differences in size distributions and circularities, where DNA-based droplets are relatively larger and more circular. These observations are consistent with previous reports on the relationship between droplet mechanical properties, morphologies, and fusion dynamics.^20,42^

**Figure 6:**
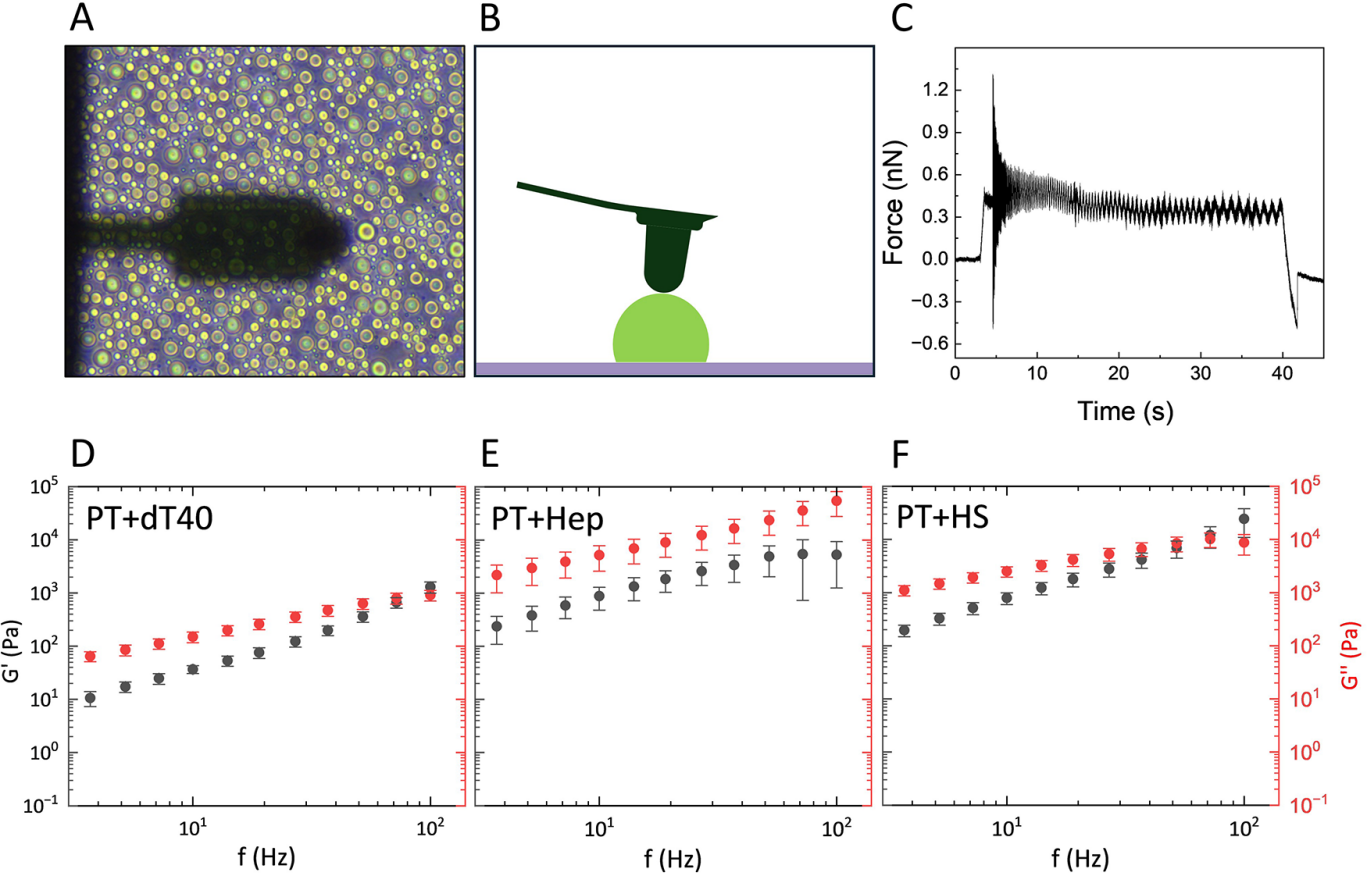
Behavior of protamine-based condensates as viscoelastic liquids. (A) Top-down view of the cantilever and measured droplet directly to the right of the cantilever tip. (B) Schematic side view of droplet (green) and cantilever (dark gray). (C) Force trace from a representative protamine-dT40 droplet. Protamine is abbreviated as PT. (D) Black (*G*′) elastic modulus and red (*G*″) viscous modulus from protamine-dT40 condensates prepared using 60 μM protamine and 30 μM dT40. Mean + SD (*N* = 7). (E) Viscoelastic moduli from protamine-heparin condensates prepared using 60 μM protamine and 30 μM heparin (Hep). Mean + SD (*N* = 8). (F) Viscoelastic moduli from protamine-heparan sulfate condensates prepared using 60 μM protamine and 15 μM heparan sulfate (HS). Mean + SD (*N* = 11).

### Protamine-Based Condensate System Successfully Loading and Releasing DNA

The above study focused on understanding the DNA-protamine interaction to shed light on sperm chromatin and fertility. The observation may, however, be exploited for engineering drug delivery vehicles. As a first step in this direction, we asked whether we could leverage programmable biomolecular condensates for spatially controlled nucleic acid delivery. To examine the viability of using protamine as a DNA delivery vehicle, a loading-and-release experiment was conducted. First, stable condensate droplets were constructed using 60 μM protamine and 30 μM heparan sulfate (Figure 7A,B, preloading). After 2 h, the droplets were treated with 30 μM fdT40 which loaded the DNA into the droplets. After another 2 h, we treated the DNA-loaded system with 30 μM heparin, which led to dramatic release of ssDNA visible in the microscopy images (Figure 7A,B, release) and was confirmed by quantification (Figure 7C,D). This treatment also led to the emergence of condensate-like structures with a distinct morphology.

**Figure 7:**
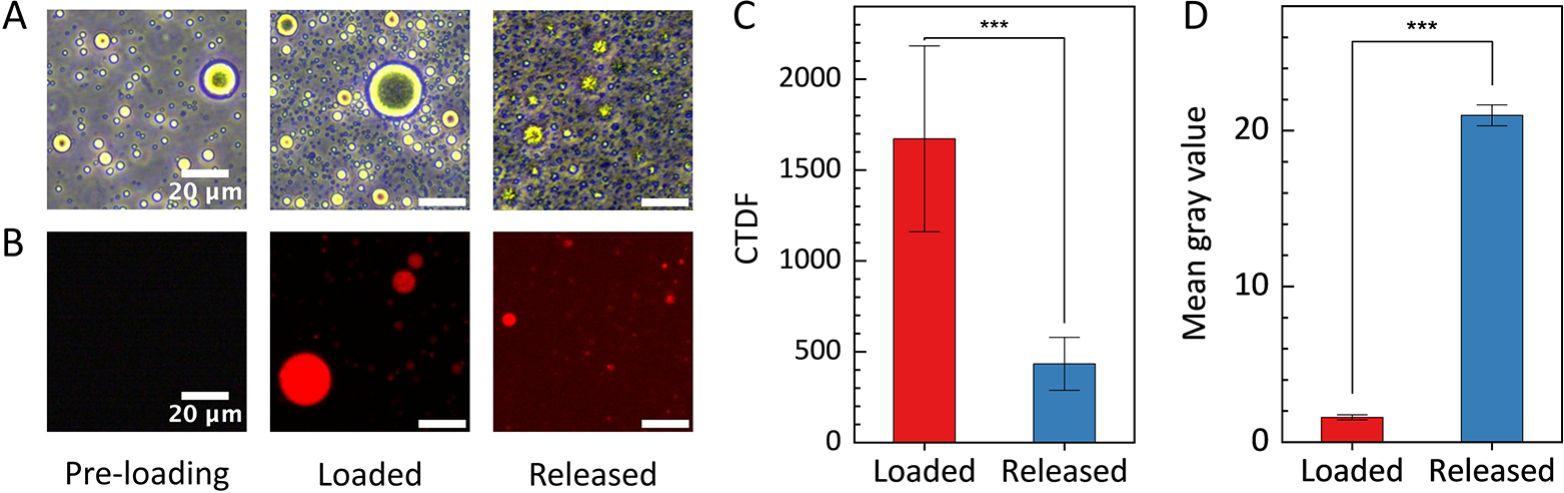
ssDNA successfully loaded into protamine-based condensates and released by using heparin. (A) Phase contrast microscopy images from in vitro droplet assay. Preloading contains 60 μM protamine and 30 μM heparan sulfate. Loading is performed using 30 μM dT40. Release is performed with 30 μM heparin. Clear morphology changes are observed after release. (B) Protamine-heparan sulfate condensates loaded using f dT40. (C) CTDF of droplets upon loading and after release. CTDF significantly decreases after release (*p* = 0.00195). (D) Mean gray value of the background upon loading and after release. The mean gray value of the background significantly increases after release (*p* = 0.00195). *p*-value styles: two-tailed, exact value, GP. *p*-values derived from Wilcoxon Signed Rank Test.

## Discussion

This study aimed to elucidate the phase separation behavior of protamine with ssDNA and dsDNA and its modulation by GAGs, to provide new insights into protamine–DNA interactions in sperm nuclei and to leverage these findings for the design of programmable biomolecular condensates for spatially controlled nucleic acid delivery. We demonstrate that protamine undergoes liquid–liquid phase separation (LLPS) with ssDNA to form liquid-like condensate droplets, whereas its interaction with dsDNA primarily results in the formation of solid-like aggregates. This contrast may be attributed to distinct binding modes and the differing physical properties of the nucleic acids: protamine binds specifically to the major groove of the rigid dsDNA helix, leading to stable aggregate formation, whereas ssDNA, due to its charge density, structure, and high flexibility, engages in more dynamic electrostatic interactions that facilitate condensate formation. Our findings reveal that protamine assembly with GAGs shares some similarities with ssDNA, albeit with clear distinctions in interaction strength and structure. We observed the formation of liquid-like condensate droplets with GAGs, exhibiting rheological properties distinct from those of ssDNA condensates. Similar to arginine-rich protamine, our data reveal that polylysine also induces the formation of condensates with ssDNA (Figure S3) and GAGs^43^ but forms aggregates with dsDNA. Polylysine carries positive charges and is known for its affinity for a minor groove of dsDNA.^44^ In contrast, other positively charged proteins, such as the H1 linker histone, form condensate droplets with both ssDNA and dsDNA.^45^ These findings underscore the influence of polymer architecture on the material properties of biomolecular condensates and align with previous observations that intermolecular interaction strength and chain flexibility are key determinants of phase-separation behavior and the viscoelastic state of the assemblies.^14,46–48^

The distinct modes of protamine interaction with DNA and GAGs enable a diverse range of state transitions (e.g., aggregate-to-condensate or condensate-to-condensate) when DNA and GAGs compete. Importantly, heparin was found to displace DNA from preformed protamine condensates or aggregates, consistent with a competitive binding mechanism driven by its high negative charge density. This displacement resulted in a transition from solid-like aggregates to liquid-like condensates in the protamine-dsDNA system and, in the case of ssDNA, facilitated DNA release and deterioration of condensate droplet morphology. HS, while structurally similar to heparin, exhibited weaker effects, consistent with its lower charge density. From a physiological perspective, these findings may shed light on heparin’s established role in sperm capacitation and chromatin decondensation during fertilization.

The potential applications of these findings extend to the design of nucleic acid delivery systems. Successful encapsulation and controlled release of ssDNA from protamine-based condensates via heparin highlight the feasibility of using protamine as a molecular carrier. Protamine has previously been used for developing nanoparticle-based delivery systems,^49^ and is known for its membrane permeabilization potential.^50^ Recent studies showed that heparan sulfate condensates can form in extracellular space and strongly interact with cells.^43^ In this context, our observation suggests a route for tissue-targeted delivery of DNA or RNA molecules, where nucleic acids can be loaded to condensates at high concentrations, while heparin can be introduced exogenously to trigger release near a permeabilized cellular membrane. Recent advances in nucleic acid delivery using particles formed via phase separation have provided approaches that can be applied to optimize the protamine-based strategy and enhance the specificity of controlled release.^51^ By tuning the length of GAGs, introducing tailored derivatives, and engineering protamines with altered charges, the delivery system and its control can be further refined.^51–53^ While future research is needed to thoroughly explore these possibilities, our current study outlines a new framework for leveraging programmable biomolecular condensates as (extracellular) release platforms for spatially controlled nucleic acid delivery.

## Conclusions

Overall, our study highlights the dynamic interplay among protamine, DNA, and charged glycosaminoglycans, offering new perspectives on the regulation of sperm chromatin compaction and decondensation. The ability of heparin to modulate protamine-driven phase separation and aggregation underscores its potential utility in reproductive medicine, particularly in assisted reproductive technologies, where controlled chromatin decondensation is critical. Further investigations of these interactions and the influence of other nuclear factors could pave the way for novel strategies to manipulate sperm-DNA packaging for therapeutic applications. Beyond this, the findings may also broadly apply to other arginine-rich proteins that can bind DNA and/or RNA and, in some cases, glycosaminoglycans, with implications for cellular RNA metabolism, viral processes, and more. Finally, this study exemplifies how engineered phase-separated condensates, with their inherently high molecular storage capacity, offer promising platforms for the sustained and highly controllable delivery of nucleic acids and other therapeutics.

## Supplementary Material



## Notes

Salt dependency of ssDNA condensation (Figure S1); magnesium effect on dsDNA/protamine aggregation (Figure S2); size distribution and morphology analysis of polylysine/ssDNA (Figure S3); protamine and hairpin loop DNA (Figure S4); salt dependency of dsDNA aggregation (Figure S5); protamine and native sperm DNA (Figure S6); phase diagram of heparan sulfate condensation (Figure S7) (PDF)
