# Supplementary material for: Protamine Drives Liquid and Solid Condensation of DNA and Glycosaminoglycans
Source: Langmuir. 2025 Sep 22;41(38):25893–902. doi: 10.1021/acs.langmuir.5c01954 (PMC12490016; doi:10.1021/acs.langmuir.5c01954)
Supplement: Supplementary file 1 [file la5c01954_si_001.pdf]

# Supplementary Information

## Protamine Drives Liquid and Solid Condensation of DNA and Glycosaminoglycans

Florian J.F. van der Harten[1,2], Vahid Sheikhhassani[1,2], Alireza Mashaghi[1,2]*
[1] Medical Systems Biophysics and Bioengineering, Leiden Academic Centre for Drug Research, Faculty of Science, Leiden University, 2333CC, Leiden, The Netherlands
[2] Laboratory for Interdisciplinary Medical Innovations, Centre for Interdisciplinary Genome Research, 2333CC, Leiden University, Leiden, The Netherlands

*Corresponding author. Email: a.mashaghi.tabari@lacdr.leidenuniv.nl

**Table of Contents:**

Figure S1. Size dependency of protamine-dT40 condensate droplets on salt concentration
Figure S2. Protamine-dA40 condensate map and characterisation
Figure S3. PolyLysine+dT40 condensate map and characterisation
Figure S4. Protamine and hairpin loop forming ssDNA
Figure S5. Aggregate formation is unaffected by salt concentration
Figure S6. Protamine assembly with sperm DNA
Figure S7. Protamine-HS condensate map

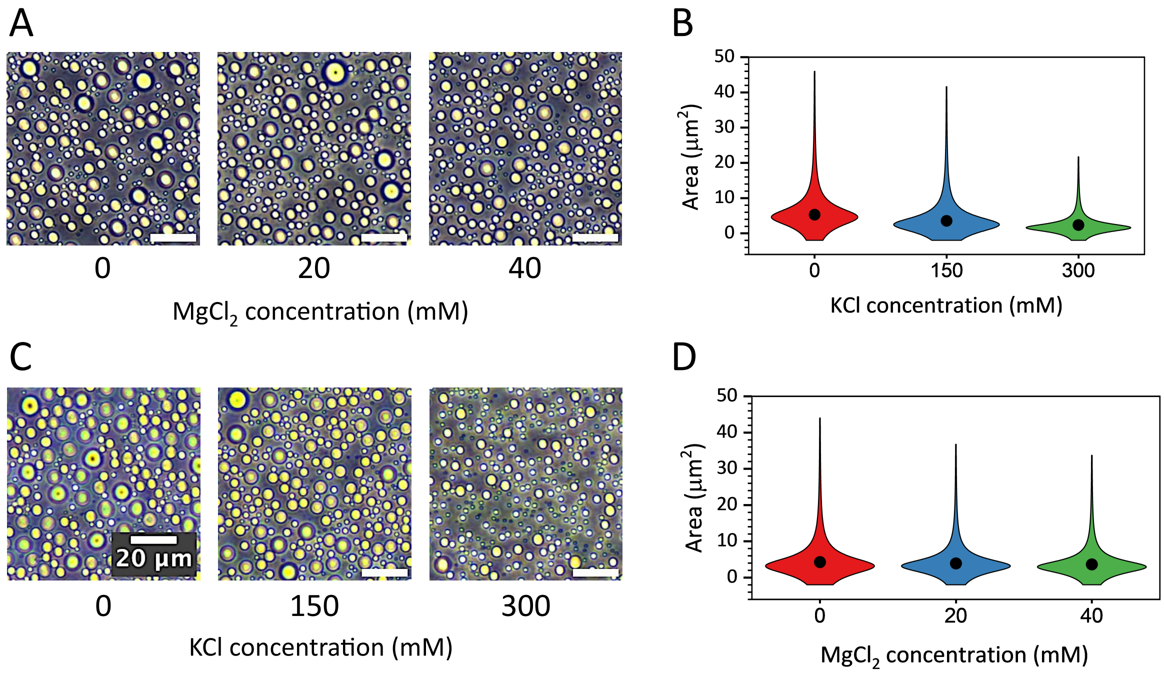


Figure S1. The mean size of protamine-dT40 condensate systems is reduced by increasing ionic strength using $MgCl_2$ and KCl. A) in vitro droplet assay images of protamine-dT40 condensates in 0, 1x, 2x physiological concentrations of KCl (pH = 7.6). B) Corresponding violin plot of size distribution of protamine-dT40 condensates in different KCl concentrations. (• = mean). C) in vitro droplet assay images of protamine-dT40 condensates in 0, 1x, 2x physiological concentrations of $MgCl_2$ (pH = 7.6). D) Corresponding violin plot of size distribution of protamine-dT40 condensates in different $MgCl_2$ concentrations. (• = mean).

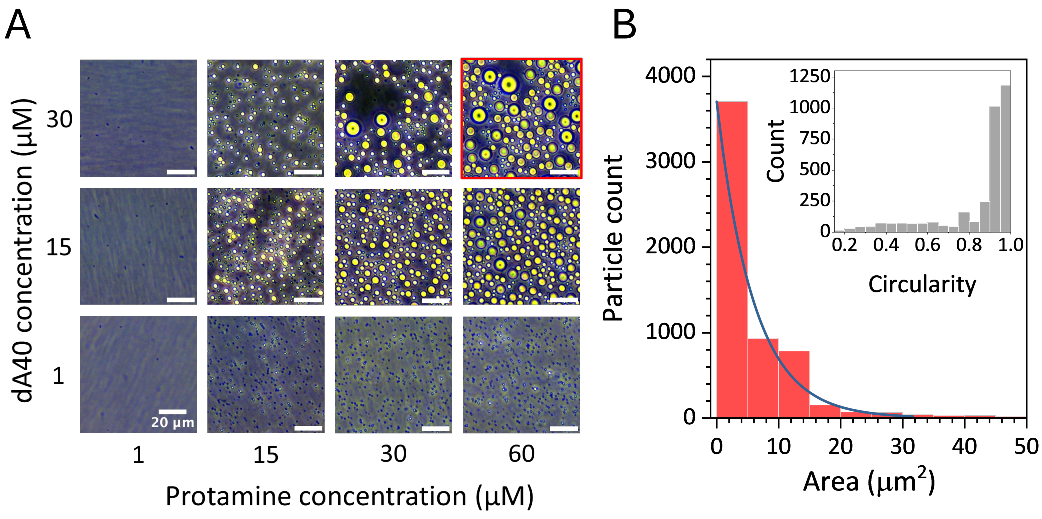

Figure S2. Protamine and dA40 phase separate to form round condensate droplets. A) phase contrast microscopy images from in vitro droplet assay of protamine-dA40 condensates (pH=7.6). B) size distribution analysis of the droplets fitted by an exponential ($y = a + be^{cx}$) curve. Inset: The droplets exhibited a mean circularity of 0.91 ± 0.2 (N = 5855), indicating their round morphology. The analysis was performed on droplets formed by 60 µM protamine with 30 µM dA40 (red outline in condensate map).

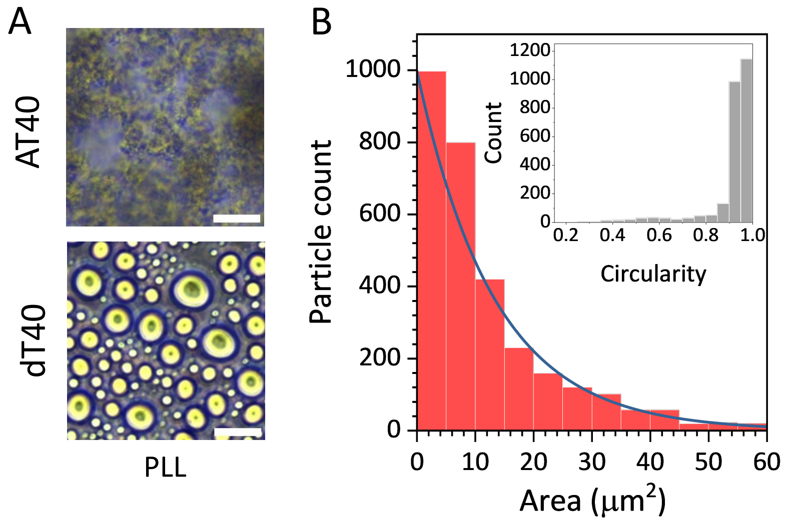


Figure S3. Polylysine forms aggregates with AT40 and phase separates with dT40 to form condensate droplets. Inset: the droplets exhibited a mean circularity of 0.93 ± 0.1 (N = 3063), indicating their round morphology.

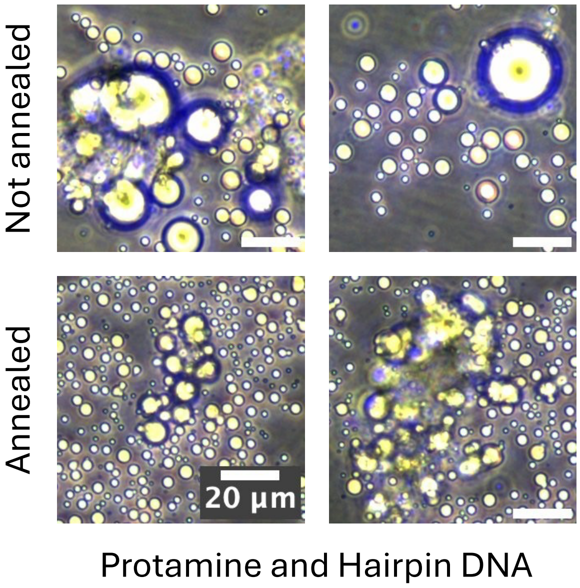


Figure S4. Protamine hairpin-forming DNA assembly. A custom hairpin DNA (5'–TTTTTTGCGCGCGCTTTTTTTTTTTTTGCGCGCGCTTTTTT–3'), designed to form a short stem-loop structure, was used in this assay. The DNA was used either directly as received from the company ("Not annealed") or after one round of melting and annealing ("Annealed"). In the

case of Annealed, the sample was heated at 95 °C for 3 minutes and then snap-cooled on ice for 5 minutes. The construct can form intra-chain loop structures or inter-chain interactions, both resulting in dsDNA regions flanked by single-stranded DNA (ssDNA) segments.

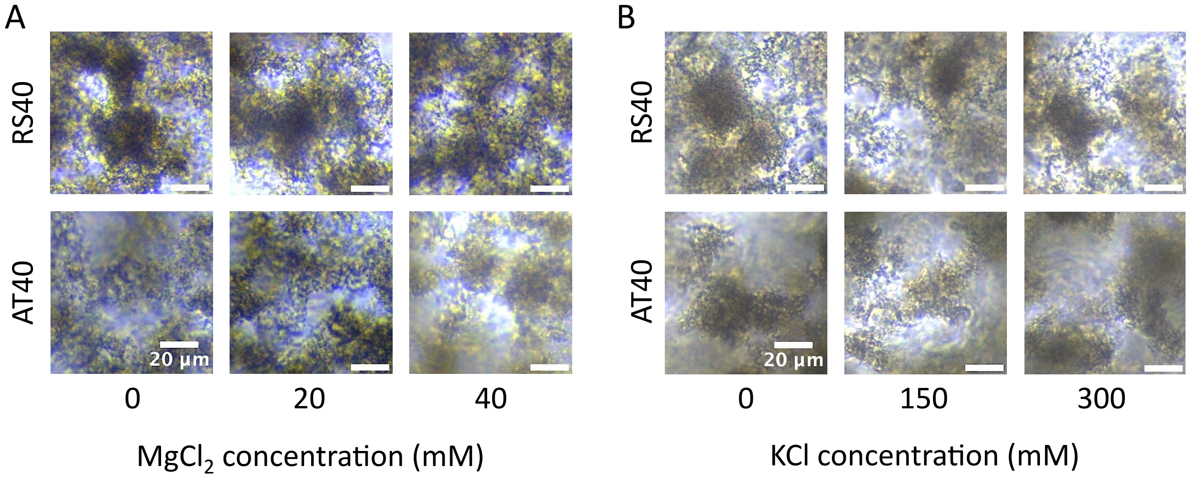


Figure S5. Aggregate formation by protamine and two constructs of dsDNA is unaffected by varying ionic strength. A) 60 µM protamine and 30 µM of AT40 & RS40 in 0, 20 and 40 mM $MgCl_2$. B) 60 µM protamine and 30 µM of AT40 & RS40 in 0, 150 and 300 mM KCl. All experiments conducted at pH = 7.6.

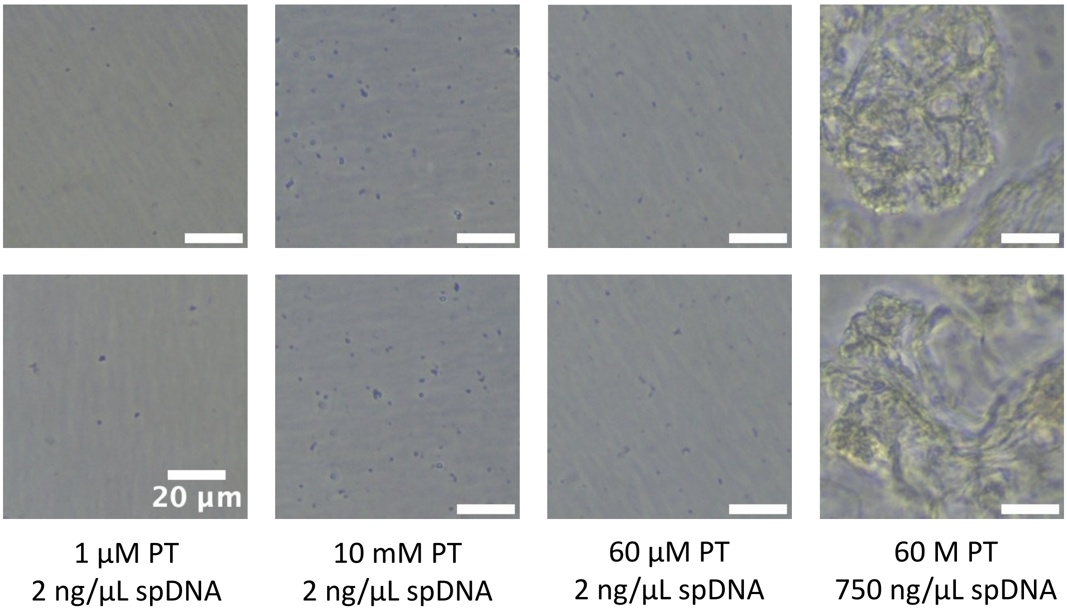


Figure S6. Protamine and native protamine sperm DNA (spDNA) in different concentrations.

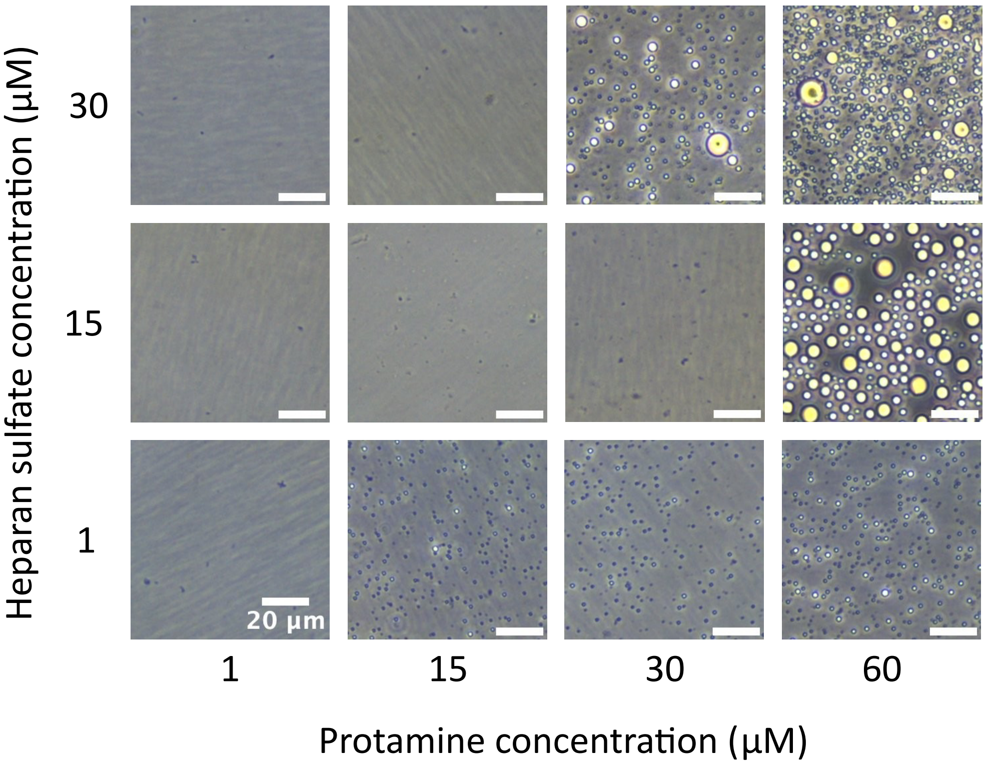


Figure S7. Protamine-HS condensate map.
